# The neuroanatomy of Broca’s aphasia

**DOI:** 10.3389/flang.2025.1496209

**Published:** 2025-02-25

**Authors:** Alexis L. Pracar, Nicoletta Biondo, Nina F. Dronkers, Maria V. Ivanova

**Affiliations:** 1 Department of Psychology, University of California, Berkeley, Berkeley, CA, United States; 2 Basque Center on Cognition, Brain, and Language, Donostia-San Sebastian, Spain; 3 VA Northern California Health Care System, Martinez, CA, United States

**Keywords:** Broca’s aphasia, language networks, brain lesions, neuroanatomy, cytoarchitecture

## Abstract

**Introduction:**

Broca’s aphasia, a condition characterized by nonfluent speech and difficulty with language production, results from focal brain damage and is most often caused by stroke. Although traditionally linked to lesions in Broca’s area (Brodmann areas 44 and 45 in the left inferior frontal gyrus), recent evidence suggests that the neuroanatomy of Broca’s aphasia is far more complex, implicating a broader network of cortical and subcortical regions. This study aimed to delineate the specific cortical and white matter features that, when damaged, lead to persistent Broca’s aphasia.

**Methods:**

39 chronic cases of Broca’s aphasia and 41 cases of stroke survivors whose language functions returned to within normal limits (WNL) were included. Lesion analyses and disconnection mapping were conducted using the Brainnetome Atlas and the Lesion Quantification Toolkit (LQT).

**Results:**

Results highlighted the critical role of the left insula, particularly its hypergranular and dorsal granular regions, which showed 99.2% and 93.6% lesion overlap, respectively, in Broca’s aphasia cases. These regions, along with portions of the motor cortex and the parietal and temporal lobes, contribute to speech production and language processing. Importantly, the traditionally defined Broca’s area showed minimal overlap, challenging the conventional understanding of its role in chronic Broca’s aphasia. In addition to cortical regions, white matter tract analysis revealed complete disconnection of key pathways, including the arcuate fasciculus, extreme capsule, and middle longitudinal fasciculus. The corticospinal tract and inferior fronto-occipital fasciculus (IFOF) were also heavily disrupted, suggesting that damage to both cortical areas and their structural connections contributes to the hallmark symptoms of Broca’s aphasia.

**Discussion:**

These findings emphasize the distributed nature of the neural network underlying Broca’s aphasia, extending beyond traditional Broca’s area to include multiple cortical regions and their associated white matter tracts. The study provides new insights into the structural basis of language impairment, offering a more nuanced understanding of Broca’s aphasia.

## Introduction

### Neural basis of Broca’s aphasia

In the United States alone, there are approximately 170,000 new cases of Broca’s aphasia per year (Ochfeld et al., 2010). Broca’s aphasia can occur after stroke and is characterized by nonfluent speech and language production with relatively less impaired comprehension. It is a complex syndrome that affects many different parts of the language network. Someone with Broca’s aphasia will struggle to name words or to produce long and grammatically correct sentences and will also experience motor speech difficulties while articulating difficult sound combinations. These each represent important pillars of communication: word finding, syntactic processing, and motor speech.

Structural features of the human brain may provide unique infrastructure for language. Lesion studies are one traditional approach in neuropsychology that can help us outline the brain areas responsible for language. This approach examines the relationship between damage to specific brain areas and the resulting language deficits, thus providing direct evidence of how various brain areas contribute to different aspects of language, such as word retrieval, sentence comprehension or production.

Cases of Broca’s aphasia, where speech and language production are impaired due to focal brain damage, offer us insights into the role of each affected region in normal language processing. Moreover, studying Broca’s aphasia through lesion analysis sheds light on the brain’s plasticity and its ability to reorganize and compensate for lost functions. This not only helps map the language network but also has the potential to inform rehabilitation and recovery following brain injury. In essence, lesion studies in cases of Broca’s aphasia serve as a valuable tool for exploring the relationships between brain structure, language function, and neural adaptability.

Lesion studies have suggested that Broca’s aphasia results from injury to multiple regions. The inferior frontal gyrus (IFG; specifically, Brodmann’s areas 44 and 45) has been traditionally associated with Broca’s aphasia, following the seminal work of Broca (1861). However, current research suggests lesions to Broca’s area do not cause chronic Broca’s aphasia, but rather a transient one (Ochfeld et al., 2010; Gajardo-Vidal et al., 2021; Mohr et al., 1978). A dissociation between Broca’s area and Broca’s aphasia is also observed in neurosurgical resection of Broca’s Area (Andrews et al., 2023). In chronic cases of Broca’s aphasia, a more widespread group of regions must be injured, specifically, the dorsolateral prefrontal cortex (BA46; BA9); supramarginal gyrus (BA40); anterior superior temporal gyrus (STG; BA22); and underlying white matter in each of these regions (Dronkers and Baldo, 2009; Fridriksson et al., 2015; Yourganov et al., 2016). Additionally, the superior precentral gyrus of the insula (SPGI, found in the anterior superior part of the left insula) is commonly infarcted in patients with apraxia of speech, a hallmark of Broca’s aphasia (Baldo et al., 2011; Chenausky et al., 2020; Dronkers, 1996; Dronkers et al., 2017; Ogar et al., 2006; Pracar et al., 2023). This previous body of literature has indicated broad cortical areas that, when injured, result in Broca’s aphasia (e.g., the IFG). What remains underexplored are the specific *neuroanatomical features* of these regions that, when damaged, result in this stereotyped constellation of symptoms.

Cortical areas do not facilitate cognition and language alone. Structural connections (bundles of axons) between different brain regions help accomplish this. It has been shown that injury to white matter tracts is necessary for chronic Broca’s aphasia (Dronkers and Baldo, 2009). Broca’s aphasia, characterized by speech and language production difficulties, is often associated with disruptions in white matter tracts that underpin the neural networks of speech and language. The arcuate fasciculus (AF) is a core language tract, whose disconnection causes impairment in both the comprehension and production of language (Bates et al., 2003; Dronkers et al., 2004; Ivanova et al., 2016, 2021; Schlaug et al., 2011). The frontal aslant tract (FAT), extending from the supplementary motor area to the pars opercularis of the IFG, is integral to motor planning and speech. For instance, apraxia of speech (AOS), a common symptom of Broca’s aphasia, is associated with disruptions in the FAT and anterior AF (Pracar et al., 2023; Zhong et al., 2022). It is of critical importance that we determine the tracts that are uniquely affected in Broca’s aphasia.

### Current approaches to mapping brain structure and function

The human brain can be conceived of as an intricate map, with individual regions demarcating distinct cognitive territories. Early mapping efforts, such as those undertaken by Korbinian Brodmann (Brodmann, 1909), delineated broad regions within this cognitive terrain. Each of these regions, like a distinct neighborhood, possessed unique characteristics based on the cytoarchitectonic structure of these cell clusters. The advent of modern neuroanatomical techniques has revolutionized our perspective of this neural landscape. As a result, we’ve transitioned from drafting rudimentary maps to creating high-resolution, detailed parcellations. This progression from rough sketches to more detailed “cartography” has redrawn borders between cognitive territories (Grill-Spector and Weiner, 2014; Weiner and Zilles, 2016; Oishi et al., 2024). Much like the boundaries between urban neighborhoods, these borders are not starkly defined but rather feature areas of overlap and crosstalk. These new maps highlight the dynamics of brain function, where areas once considered hyperspecialized are now understood to host multiple functions and engage in complex interactions with other regions. For example, newer atlases like BigBrain (Amunts et al., 2013), Human Connectome Project (HCP; Glasser et al., 2013), and the Julich-Brain (Amunts et al., 2020) mark significant advances in our understanding of cytoarchitecture, myeloarchitecture, and receptor architecture.

In particular, the Brainnetome atlas (Fan et al., 2016) moves beyond purely local microstructural features like the early 20th-century Brodmann areas to incorporate functional features. The Brainnetome atlas integrates data from resting state functional MRI, structural MRI, and diffusion MRI, encompassing both the brain’s physical structure and its functional dynamics. This atlas transforms traditional anatomically defined brain regions into functionally distinct parcels that reflect their unique structural and functional connectivity profiles. The Brainnetome atlas also considers long-range neural connections, aligning with the modern understanding that cognition emerges from network-level interactions. This atlas is consistent with state-of-the-art cytoarchitectonic findings on microstructural heterogeneity within brain regions on the level of cyto- and receptor architecture (Zilles and Amunts, 2018). A detailed cytoarchitectonic investigation of cortical areas associated with Broca’s aphasia has not previously been undertaken. The Brainnetome atlas, however, is a purely cortical parcellation. To better investigate how white matter fiber pathways are affected by lesions, complementary white matter disconnection analyses are needed. In the absence of original diffusion tensor imaging (DTI), white matter disconnection can be investigated through indirect structural disconnection mapping (Sperber et al., 2022). The Lesion Quantification Toolkit (LQT; Griffis et al., 2021) is one of the available toolboxes that embeds the lesion(s) into a standard atlas of white matter tracts to estimate the disconnection to individual fiber tracts. It was selected to provide complementary insights into the white matter fiber pathways that are affected by the lesions of participants with Broca’s aphasia.

### The current study

The current study investigated 39 cases of Broca’s aphasia to ask the following research questions: (1) What are the injured cortical regions and white matter tracts that result in persisting Broca’s aphasia? (2) What is the cytoarchitectural landscape of these regions?

## Materials and methods

### Participants and measures

Participants included 39 stroke survivors with chronic Broca’s aphasia and 41 who tested within normal limits (WNL; meaning that an individual had a stroke that resulted in aphasia initially but resolved to performance within normal limits on the Western Aphasia Battery). The Western Aphasia Battery (Kertesz, 2007) was used to assess speech and language abilities and to determine aphasia subtypes. All participants had sustained a single left hemisphere stroke at least 6 months before being tested and scanned. All participants were right-handed, had native-like proficiency in English prior to their stroke, had more than 8 years of education and normal or corrected-to-normal hearing and vision. All participants provided their informed consent, and research was approved by the Institutional Review Boards at the VA Northern California Health Care System and the University of California, Berkeley in line with the Helsinki Declaration. Demographic data and average WAB subtest scores for the 39 Broca’s and 41 WNL participants are reported in Table 1.

### Neuroimaging data

#### Neuroimaging data acquisition

The lesions of the participants were manually delineated based on MRI or CT scans obtained at least 6 months post-stroke, in the chronic stage. For comprehensive details on the procedures used in earlier scans for data collection (n = 79) lesion delineation, and normalization, refer to Baldo et al. (2013) (as a subset of the data from this study was used in the current study). Post-2012 data collection utilized either a 3T Siemens Verio (*n* = 79) or Trio (*n* = 2) scanner with an MPRAGE sequence, with a voxel size of 1 × 1 × 1 mm.

#### Manual lesion segmentation

Using MRIcron software (Rorden, 2007) or ITK-SNAP (Yushkevich et al., 2006), lesions were manually outlined on each patient’s native T1-weighted images. Where available, T2-weighted and FLAIR images were aligned with T1 images to confirm lesion boundaries. For normalization to the MNI template, we used a modified SPM8 algorithm with lesion-specific cost function masking (part of the SPM8′s Seg toolbox). This algorithm was tailored to enhance normalization of the ventricles and deep white matter, employing an age-appropriate template and a head model. This approach ensured a more accurate fit to the template without altering the overall brain structure. Lesion masks were represented in MNI space with a resolution of 1 mm isovoxels. Subsequent lesion overlays were created using FMRIB Software Library (FSL; Jenkinson et al., 2012).

#### Creation of lesion overlays

To isolate the voxels most commonly lesioned in patients with Broca’s aphasia, we overlapped the individual lesions of all Broca’s aphasia cases. To determine the commonly-affected areas, we thresholded the resulting overlay to only include those voxels in which 80 percent of individuals had a lesion. The threshold of 80 percent was chosen because it highlights the core of the lesion site in the cohort of participants with Broca’s aphasia. Henceforth, we will refer to this thresholded overlay as the **common Broca’s aphasia lesion**. Then using this thresholded lesion overlay (common Broca lesion) as a single ROI (with 0 indicating that the voxel is spared, and 1—completely damaged), we determined the percent of overlap with each parcel in the Brainnetome atlas. This analysis allowed us to consider the neuroanatomical features within the regions most commonly affected in Broca’s aphasia.

#### Creation of the subtraction map

The overlapped area emerged with a central area within the distribution of the middle cerebral artery. It could be construed that the regions most commonly lesioned in the cohort of participants with Broca’s aphasia were merely a by-product of the brain’s vasculature, commonly lesioned following any left hemisphere stroke, thus not specific to Broca’s aphasia *per se*. Additionally, strokes that result in a Broca’s aphasia are normally quite large and occur in these stereotyped patterns of the middle cerebral artery. It was thus necessary to compare the lesions of patients with Broca’s aphasia with other patients who also had left hemisphere strokes but were no longer aphasic. The subtraction map was created using lesions that did and did not result in Broca’s aphasia to account for regions that may simply be injured to the size and probable locations of stroke. Thus, lesions from participants classified as WNL according to the WAB (Kertesz, 2007), were compared to those with Broca’s aphasia to rule out any common areas of infarction that were not related to Broca’s aphasia. First, we overlapped the lesions for each group (Broca and WNL) separately as described above, and for each voxel calculated the proportion of participants with a lesion in that voxel (i.e., number of lesions overlapping in that voxel divided by the number of participants in each group). Second, we subtracted these two maps (Broca cohort voxels minus WNL cohort voxels; e.g., 0.7 minus 0.1 for a voxel that is lesioned in 70% of the Broca cohort, but only lesioned in 10% of the WNL cohort, resulting in a value of 0.6).

The resulting **subtraction map** contained values for each voxel ranging from −0.19 to 0.85, that indicated the prevalence of lesions in the Broca cohort relative to the WNL cohort, with positive values highlighting voxels that are more commonly lesioned in the Broca’s patients. However, given the lower percentage of lesion overlap in the WNL group, we decided to use a more rigorous threshold to highlight the areas truly specific to the Broca cohort. The maximum overlap value for voxels in the Broca’s cohort was 0.97, meaning that the highest area of overlap in this cohort contained voxels in which 97% of patients had a lesion. The maximum overlay value for voxels in the WNL cohort was 0.42. We defined the threshold as the difference between the maximum overlap voxel values for the Broca cohort (0.97) and WNL cohort (0.42), which was 0.55. This stringent threshold is what allows us to account for any disparities in lesion size between the two groups. This method helps identify voxels with lesion prevalence meaningful for understanding Broca’s aphasia, as they significantly vary from the lesion distribution seen in the WNL cohort. For an additional step-by-step explanation, see Supplementary material.

Thus, we thresholded the voxels in the subtraction map to include only those with values of 0.55 and above, to outline areas that were substantially more lesioned in the Broca’s cohort above and beyond what could be expected simply due to the initially higher lesion overlap in the Broca’s cohort. Then this subtraction map was binarized and used in the Brainnetome atlas analysis. This way, we could understand which regions were uniquely more lesioned in patients with Broca’s aphasia even when we accounted for areas commonly lesioned after left hemisphere stroke.

### Identifying involvement of gray and white matter areas

#### Brainnetome atlas

The Brainnetome atlas (Fan et al., 2016) was selected due to its comprehensive mapping of the brain based on multimodal data. This atlas was created from multimodal imaging of 40 healthy control brains. What follows is a description of the pipeline for the creation of the Brainnetome atlas: An initial parcellation of each structural scan is made using a standard anatomical atlas [Desikan–Kiliany (DK) atlas; Desikan et al., 2006], to define regions of interest (ROIs). These ROIs serve as seed masks for further analysis. Multimodal MRI data, including structural MRI, diffusion MRI (dMRI), and resting-state functional MRI (rfMRI), are then integrated for each of these ROIs. Structural MRI gives the anatomical details, diffusion MRI maps the white matter fiber pathways that stem from the ROIs, and resting state fMRI shows functional connectivity patterns. This integration allows for a comprehensive view of both the structural and functional architecture of the brain. Probabilistic diffusion tractography, using dMRI data, is used to trace the neural connections emanating from each ROI. This step is crucial to understand the physical axonal connections of each region. Then the resting state connectivity data (from fMRI) is used to subdivide the initial ROIs into smaller, more functionally homogeneous parcels. This is achieved through connectivity-based parcellation, where algorithms cluster voxels within a ROI based on similarities in their connectivity profiles. The outcome of this is a set of new, refined parcels that are defined not just by their anatomical location but also by their distinct connectivity patterns. These parcels are labeled differently from their original anatomical names but do retain some of the original nomenclature (e.g., BA44 is broken into dorsal and ventral components called 44d and 44v to reflect their unique structural and functional characteristics).

In the current study, we used Python to quantify the overlap between the brain lesion overlay for the Broca’s cohort, the subtraction map, and Brainnetome atlas regions. Key libraries used included *nibabel* (Brett et al., 2020) for handling neuroimaging data, *numpy* (Harris et al., 2020) for array operations, and *pandas* (McKinney, 2010) for data management. We loaded lesion images and the Brainnetome atlas using *nibabel*, and a lookup table and participant data using *pandas*. The overlay was first co-registered with the Brainnetome atlas dimensions.

#### Indirect structural disconnection mapping

Finally, we wanted to examine white matter contributions to the unique cases of Broca’s aphasia, as it is well known that white matter connectivity is crucial for language. The Brainnetome atlas is a purely cortical parcellation, although it considers structural and functional connectivity, it does not directly state which white matter pathways are affected by the lesions. To address this, we used the Lesion Quantification Toolkit (LQT; Griffis et al., 2021). Indirect mapping of structural disconnection can identify disrupted fiber pathways in brain-injured individuals by integrating spatial lesion data with normative data on white matter anatomy. This method embeds lesion data into a standard white matter connectome to determine which white matter structures, and to what extent, might be affected by this specific lesion. In this study, LQT was employed to assess white matter tract disconnections using the HCP-842 atlas (Yeh et al., 2018). This atlas was developed from high-resolution diffusion MRI data gathered from 842 healthy individuals. The common Broca’s aphasia lesion and the subtraction map were loaded into LQT to determine the white matter pathways affected by the lesions.

## Results

### Cytoarchitectural features of the injured cortical regions that resulted in persisting Broca’s aphasia

#### The common Broca’s aphasia lesion

**FIGURE 1:**
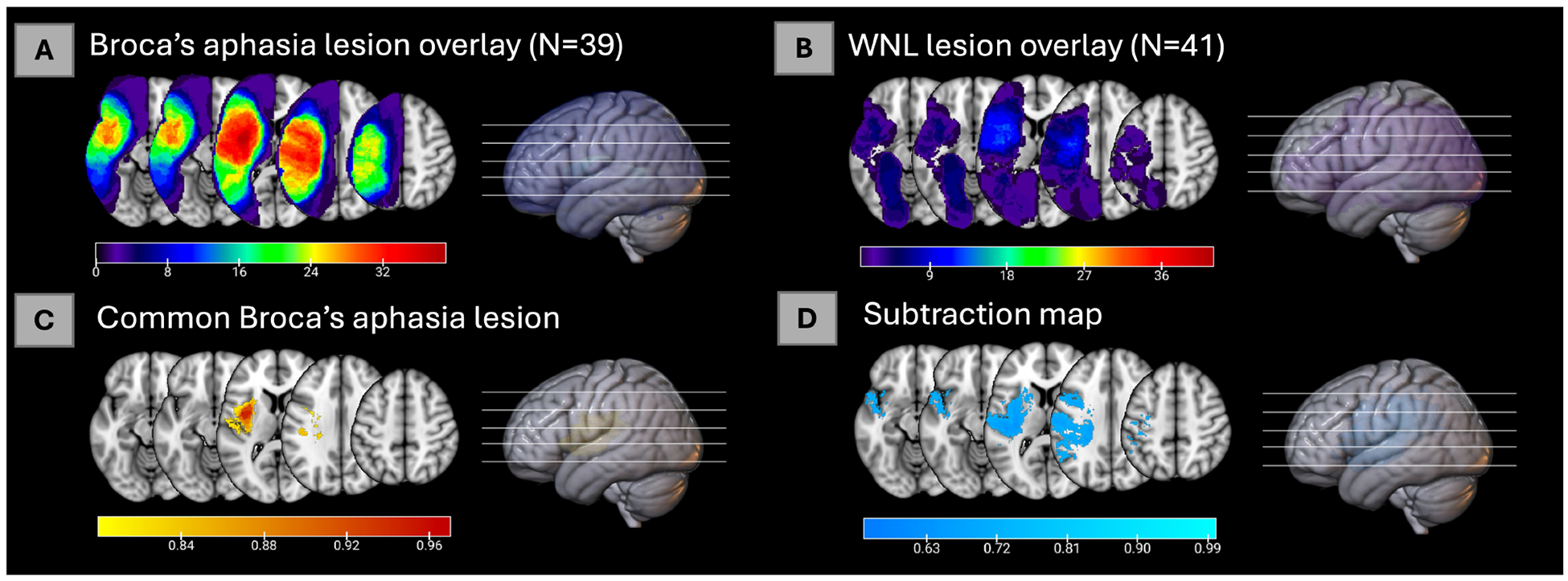
**(A)** Lesion overlay of 39 cases of Broca’s aphasia. **(B)** Lesion overlay of 41 cases of within normal limits participants [for **(A, B)**, color bars represent how many individuals have a lesion in each voxel]. **(C)** The common Broca’s aphasia lesion in 80% of the 39 cases of Broca’s aphasia [for **(C)**, color bar represents proportion of individuals lesions in each voxel]. **(D)** Subtraction map of lesions of Broca’s aphasia minus WNL lesions thresholded at 55% [for **(D)**, color bar represents percent voxels resulting from the subtraction map, see Methods for details].

**FIGURE 2:**
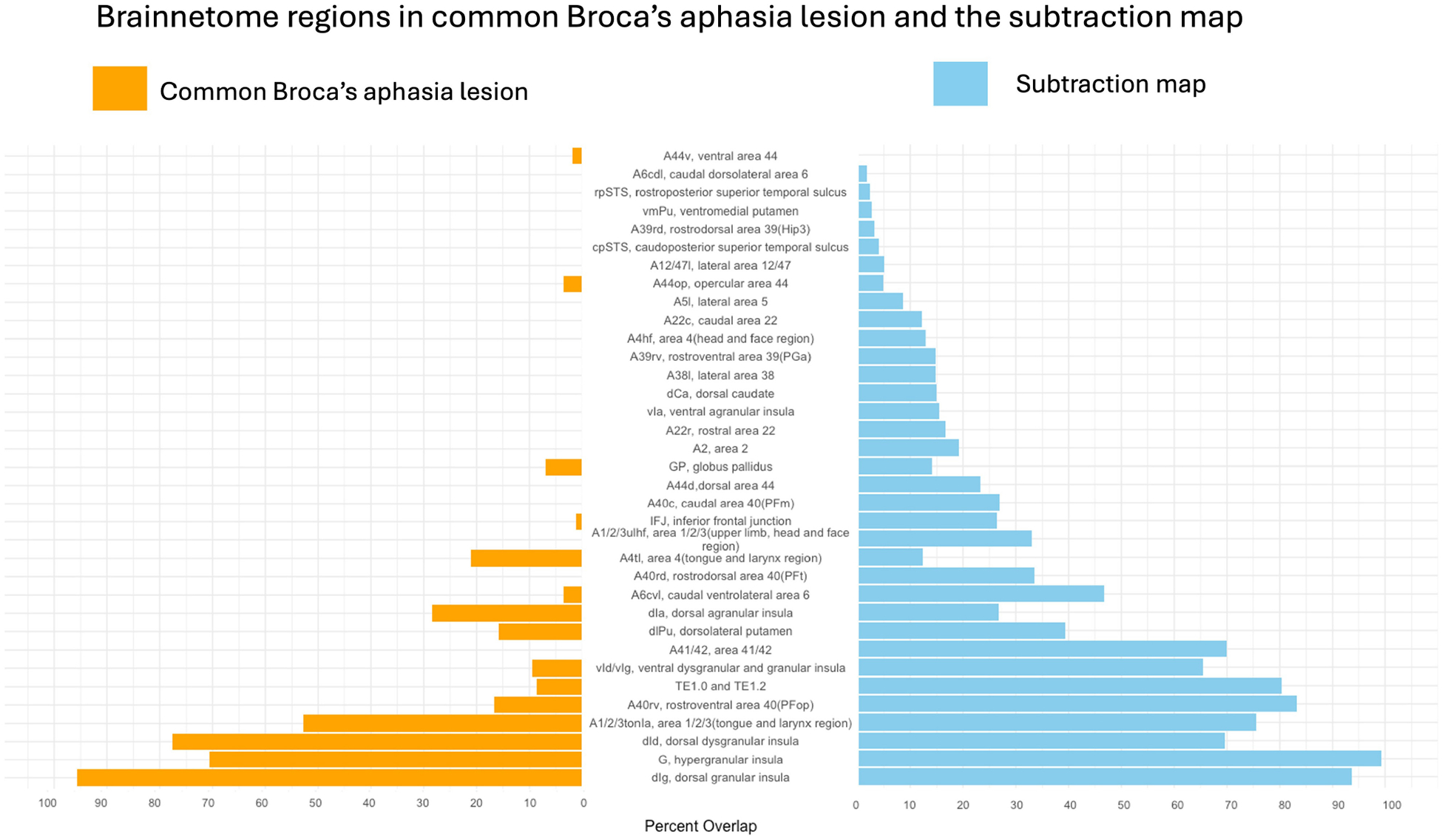
Areas from the Brainnetome Atlas overlapping with the common Broca’s aphasia lesion and the subtraction map. Percentage of lesion overlap within specific brain regions for the common Broca’s aphasia lesion is shown in orange. Regions of overlap for the subtraction map are shown in blue. The dorsal granular insula (dIg) showed the greatest overlap.

First, lesion overlays for all the participants in the Broca’s and WNL cohorts were created and can be seen in Figures 1A, B respectively. Then the common Broca’s aphasia lesion (see Figure 1C) was created by thresholding the Broca’s lesion overlay at 80%; this map included insular, frontal, parietal, and temporal regions. Figure 2 indicates that critical overlaps were observed in the left insula, with the dorsal granular insula at 95.7%, dorsal dysgranular insula showing a 77.5% overlap, hypergranular at 70.6%, and agranular portions at 28.2%, per the Brainnetome Atlas. Frontal regions, particularly those controlling tongue and larynx movements, had overlaps of 52.9% and 21%, respectively. Traditional Broca’s area, BA44 and BA45, showed lower overlaps (3.5%, 1.8%, and 0.3% for BA44 subdivisions within the Brainnetome atlas; 0.00 % for both BA45 subdivisions). In the parietal and temporal lobes, A40rv and the primary auditory cortex regions (TE1.0 and TE2.0) showed 16.6% and 8.9% overlaps, respectively.

#### The subtraction map

The subtraction map (see Figure 1D) was used to compare lesion prevalence between Broca’s aphasia and WNL cohorts and to control for common lesion-sites due only to the vasculature of the middle cerebral artery. A threshold was applied to highlight regions more commonly affected in Broca’s patients. The resulting map was then binarized and analyzed to identify brain areas uniquely associated with Broca’s aphasia. The map was used to confirm that the regions identified in the common Broca’s aphasia lesion were not merely a consequence of the middle cerebral artery’s distribution but rather specific to the symptoms of Broca’s aphasia. The left insula emerged as a critical region in both analyses, particularly in its dorsal granular and dysgranular regions, which showed significant overlap in the common Broca’s aphasia lesion (95.7% and 77.6%, respectively) and substantial overlap in the subtraction map (99.2% and 93.6%). This consistency reinforces the insula’s pivotal role in Broca’s aphasia. Traditional Broca’s area (BA44 and BA45) (23.4% for BA44d, 0.8% for BA44v, none for BA45), showed lower overlap percentages in both analyses while regions such as the rostral ventral area 40 (A40rv) (83.3%) and the primary auditory cortex (TE1.0 and TE2.0) (80.4%) also exhibited consistent overlap. This alignment between the common Broca’s aphasia lesion and the subtraction map results underscores the specificity and relevance of these regions in understanding the neural basis of Broca’s aphasia, rather than being a mere function of arterial distribution.

#### White matter tracts also contributing to persisting Broca’s aphasia

**FIGURE 3:**
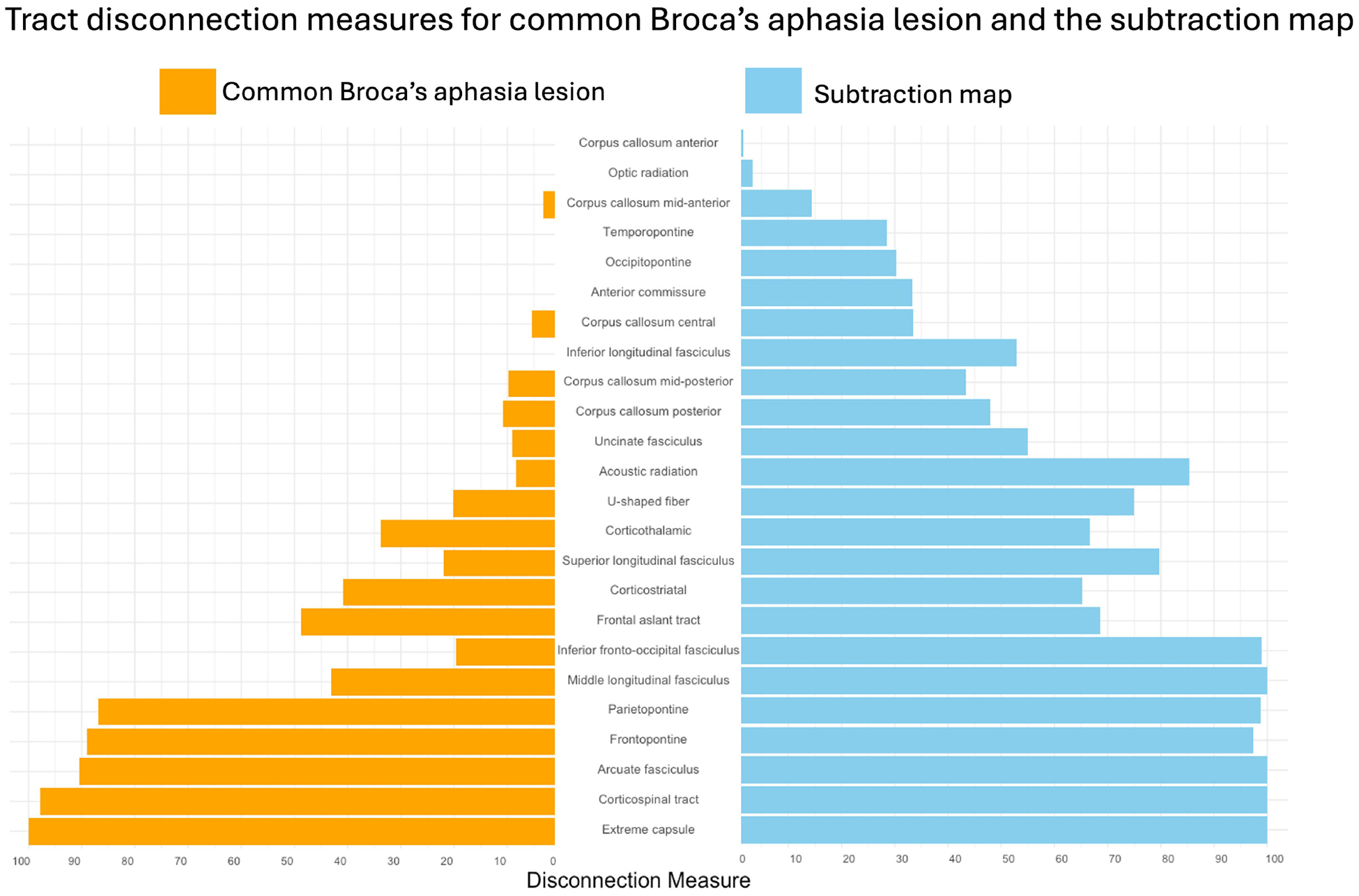
Tract disconnection measures for the common Broca’s aphasia lesion and the subtraction map. Percent of disconnection of white matter tracts in individuals with Broca’s aphasia, as defined by the common Broca’s aphasia lesion, shown in orange. Percent of disconnection of white matter tracts that are injured more commonly in Broca’s aphasia when compared to WNL, are shown in blue. The results were obtained by loading the common Broca’s aphasia lesion and the subtraction map into the LQT (Griffis et al., 2021).

The subtraction map indicated multiple white matter tracts associated with Broca’s aphasia. The arcuate fasciculus (AF) showed a complete disconnection, with a disconnection index of 100.0 percent (see Figure 3). Similar complete disconnection (100 percent) was observed in the extreme capsule (EC) and the middle longitudinal fasciculus (MdLF). The corticospinal tract showed a near-total disconnection with an index of 99.96 percent. Additionally, the inferior fronto-occipital fasciculus (IFOF) showed a 98.86 percent disconnection index. The parietopontine tract demonstrated a 98.7 percent disconnection index. Finally, the frontopontine tract showed a disconnection index of 97.4 percent. These tracts were reflected in the subtraction map, confirming their uniqueness to Broca’s aphasia.

## Discussion

The subtraction map identified multiple gray matter regions associated with Broca’s aphasia. The left insula emerged as most critical, with the commonly affected area overlapping by 99.2% in the hypergranular insula, 93% in the dorsal granular insula, 69.5% in the dorsal dysgranular insula, 65.5% in the ventral dysgranular an granular insula, and 26.7% in the dorsal agranular insula, highlighting the importance of these areas in language processing and speech production. The motor cortex, particularly the regions associated with the tongue and larynx, overlapped with the subtraction map by 75.6%. The regions traditionally part of “Broca’s area” (A44d, A44v, A44op, A45r, A45c), overlapped all below 5%, apart from 23.4% in A44d, challenging the conventional focus solely on Broca’s area. Additionally, parietal and temporal lobes exhibited involvement, with 83.2% overlap in the A40rv region, 69.9% overlap in A41/42 (primary auditory cortex, bordering Wernicke’s area), and 80% overlap in areas TE1.0 and TE2.0, adjacent to primary auditory cortex. White matter tracts like the AF, EC, and MdLF showed complete disconnections, underscoring the integral role of white matter pathways. Overall, the findings suggest that Broca’s aphasia results from disruptions in a complex network involving insular, frontal, parietal, and temporal regions, along with underlying white matter pathways. Below we discuss regions that were identified in both maps, reporting the specific percentages from the subtraction map, to focus on the unique contributions of these regions.

### Insular regions

The highest regions of overlap in Broca’s aphasia were centered in the left insula. The **dorsal granular insula** (93.6%) contains thinner layers II and IV, with large pyramidal cells in layer V. The thinner layers II and IV may allow for denser synaptic features. The large pyramidal cells in layer V may perform computationally intensive work, as these same cells are found in layer III and V in the higher order IFG. The left dorsal granular insula (dlg) is functionally connected to portions of the frontal lobe and the temporal lobe (medial and lateral). Specifically, the dlg insula is connected to the anterior and posterior superior temporal sulcus (aSTS, pSTS) as well as the superior temporal gyrus (STG). In the frontal lobe, the dlg insula has connectivity with almost all components of “Broca’s Area” (BA44, BA45) as reimagined by the Brainnetome parcellation (A45c, A45r, A44v), with the notable exclusion of A44d (the dorsal component of A44). In the precentral gyrus, the dlg insula is connected with A4tl, which is the motor representation of the tongue and larynx. The dlg insula is also connected to the entirety of the medial temporal lobe. The collection of regions with which the dlg insula is connected is from the language network, however, the dlg insula region itself is implicated in a wide set of functional tasks (interoception, perception/somatosensation, disgust, pain processing, discrimination tasks) (Kurth et al., 2010). The **dorsal dysgranular insula** (69.5%) contains decreased cortical thickness and increased myelination of granular layers II, IV, and V. The increased myelination in these layers could serve to increase processing speed and efficiency, which may be needed for language processing and speech production. In a meta-analysis of 1,700 studies, Kurth et al. (2010) found the mid dysgranular insula to be associated with language and motor tasks. An additional feature of the dysgranular cortex in the insula is the presence of Von Economo Neurons (VENs) in anterior layer V (Morel et al., 2013). This region also contains the SPGI, which has been implicated in apraxia of speech, a hallmark symptom of Broca’s aphasia (Baldo et al., 2011; Chenausky et al., 2020; Dronkers, 1996; Pracar et al., 2023). The **hypergranular insula** (99.2%) contains dense layers II and IV, with large pyramidal cells near layer IV. The increased number of neurons in layers II and IV may reflect the terminations and origins of long-range connections needed for speech and language. The hypergranular (G) insula, has a connectivity profile more like the dorsal granular insula than the dorsal dysgranular insula. In addition to the same connectivity with the temporal and parietal lobes, the G insula has connectivity to the IFG but not the MFG and SFG. Functionally, the G insula has been shown to be involved in perception and somatosensation, gustation, as well as monitoring and discrimination paradigms, which makes its function more like the dld. Though not as commonly lesioned in the Broca’s cohort (per the subtraction map) the **dorsal agranular insula** (overlap 26.6%) contains the highest relative density of VENs in layer V and a complete lack of granular cells. The agranular insula also contains columnar arrangement of the neurons in layer III and V, thought to originate from the bulky columnar layout of the VENs in layer V. The columnar organization of neurons is a distinct architectural feature of certain brain regions. This alignment may facilitate efficient local processing and rapid intracolumnar communication. Neurons within a column are often functionally similar and can process specific types of information more efficiently. For example, the columnar organization in the occipital cortex has been shown to facilitate visual processing (Mountcastle, 1997). Particularly, selectivity for specific orientations of visual stimuli can be localized to individual cortical columns. Finally, the **ventral dysgranular and granular insula** (overlap 65.5%) has also been, in humans, to contain VENs. This extension of the VENs, beyond agranular cortex is unique to the human brain (Morel et al., 2013).

Von Economo neurons (VENs) are distinct in their large fusiform or spindle-shaped structure, characterized by elongated cell bodies (Watson et al., 2006). This unique morphology sets them apart from other neuron types. The bipolar nature of these neurons, with symmetrical apical and basal dendrites, allows for efficient and balanced signal integration from different parts of the brain. This symmetrical structure might be crucial for the rapid processing and transmission of complex information. Interestingly, VENs predominantly develop postnatally, with significant growth occurring within the first 8 months of life, as noted by Allman et al. (2011). The late development of VENs could indicate their involvement in higher-order cognitive processes that mature postnatally, such as aspects of speech and language. VENs are hypothesized to facilitate the rapid comparison of inputs, a function that could be essential in intuitive decision-making and complex social cognition, and possibly even speech production (Watkins et al., 2002). Specifically, their ability to quickly integrate diverse sensory and cognitive inputs might be critical to the rapid switching of motor commands. In the context of Broca’s aphasia, where there is a disruption in speech production and language, the role of VENs could be particularly pertinent. The influence of VENs on the columnar arrangement in the insula could be pivotal for processing complex information like linguistic cues and social communication. The columnar organization, facilitated by the morphology of VENs, might support specialized processing streams within the insula. Input into a column of neurons is sent vertically, rather than horizontally, as demonstrated by Hubel and Wiesel (1997). Nimchinsky et al. (1999) showed that axons from the VENs supply the anterior cingulum bundle in the anterior cingulate. This specialized processing, with connections spanning the cortex, could be essential for the rapid integration of language production processes with other cognitive domains (Watson et al., 2006). Understanding the potential role of VENs in cognitive processing can provide novel insights into the neural underpinnings of Broca’s aphasia. Further research into the specific contributions of VENs in neural circuits related to language and social communication could reveal new therapeutic targets for aphasia and related disorders.

The insula, particularly the granular portion is more commonly lesioned in participants with Broca’s aphasia. The columnar arrangement, combined with the unique properties of VENs, might be critical in coordinating the neural circuits involved in speech production. In Broca’s aphasia, where speech production is hindered, disruptions in this columnar arrangement or in the functioning of VENs could lead to inefficiencies in neural signaling and integration. This could manifest as difficulty articulating words, despite often retaining comprehension abilities.

### Frontal regions

Several regions in the lateral frontal cortex were implicated in Broca’s aphasia. The **tongue and larynx regions in the ventral precentral and postcentral cortex (A1/2/3tonla)** were identified (75.6%). These regions have also been implicated in apraxia of speech (Itabashi et al., 2016) and in neurosurgical patients with lasting Broca’s aphasia (Andrews et al., 2023). In a VLSM study of 289 patients, 19 who had postoperative Broca’s aphasia, injury to the ventral sensorimotor areas and supramarginal gyri was correlated with the language disorder (Andrews et al., 2023). Additionally, reduced fluency scores were observed with increasing resection of precentral, postcentral, supramarginal voxels within the significant VLSM cluster. While these regions may be important for coordinating complex articulatory movements, as impacted in apraxia of speech, they are also needed for production of even the simplest words. This may explain why people with Broca’s aphasia tend to have problems speaking aloud words for which they can demonstrate comprehension. In the postcentral gyrus of the parietal lobe, the tongue and larynx region contains somatosensory representation of these parts of the articulatory system. Whereas representation in the premotor strip is necessary for the motor coordination and action of these muscles, the postcentral representation is needed for proprioception. In fact, this region shares connectivity with A4tl, which is the executor of motor commands, suggesting that they work in tandem for successful use of the larynx and tongue, which must be tightly coordinated for expression of language through vocal speech. Additionally, this region has connectivity with the IFG, and the pSTS, aSTS, STG, and the IPL. Direct connections between motor control and sensory feedback for articulators may facilitate language expression in humans. Though not explicitly detailed in the Brainnetome parcellation, these regions synapse directly onto motor neurons present in the tongue and larynx, which is thought to be needed for complex vocalizations. Another example of complex vocalizers are songbirds. In songbirds, the high vocal center (HVC) contains the motor sequence code, which projects to the robust nucleus of the arcopallium (RA), which is involved in motor execution. Direct projection of RA to vocal motor neurons (syrinx muscles) is like the human projection from motor face area to brainstem nucleus ambiguous neurons to muscles of the larynx (Hahnloser et al., 2002; Jarvis, 2013). Nonhuman primates lack this connection. It should be noted that only the postcentral representation of tongue and larynx was unique to those with Broca’s aphasia. This is likely because some in the WNL cohort had residual apraxia of speech in the absence of language problems.

In the current study, injury to Broca’s area (traditionally defined as Brodmann’s areas 44 and 45) was not associated with Broca’s aphasia. Per the subtraction map, within the territory of Broca’s area, we see minimal damage to ventral, opercular, and dorsal areas 44 (overlap 0.8%, 4.9%, and 23%), which contain dysgranular layer IV, distinguishing them from rostral and caudal 45 (overlap 0.3% and 0.2%). In the common Broca’s aphasia lesion, the percentages are also low (A44op 3.5%, A44v 1.8%, A44d 0.3%, and all of the A45 components at 0%). Injury to the IFG may not be the source of a persisting Broca’s aphasia. While this may seem surprising, other work has shown that lesions to or resections of BA44/45 do not lead to long-lasting language deficits (Ochfeld et al., 2010; Gajardo-Vidal et al., 2021; Andrews et al., 2023). Ochfeld et al. (2010) found only transient Broca’s aphasia was caused by focal lesions to Broca’s areas in a cohort of 50 acute patients. In a study of 134 stroke survivors (Gajardo-Vidal et al., 2021), it was shown that injury to Broca’s area was not associated with long term speech production deficits, rather the underlying white matter around the insula and in the anterior AF was implicated. In 289 neurosurgical patients (Andrews et al., 2023), resection of Broca’s area did not cause a lasting Broca’s aphasia, rather, resection of the precentral, postcentral, supramarginal gyri were associated with the symptoms of Broca’s aphasia. Finally, in another study of 334 patients with post-stroke aphasia (Wilson et al., 2023), lesions restricted to the frontal lobe showed impairment mainly in the acute phase which was largely resolved by the chronic phase. The notion that Broca’s area is involved with Broca’s aphasia could be because Broca’s area is commonly lesioned alongside the regions we find to be more often related to Broca’s aphasia. The precentral regions, for example, are found in the common Broca’s aphasia lesion, a region immediately posterior to Broca’s area. The insula emerged as the most significantly lesioned area in the common Broca’s aphasia lesion, specifically, 95.7% in the dorsal granular insula, 77% within the dorsal dysgranular insula, and 70.6% in the hypergranular insula. The insula is directly mesial to Broca’s area. Likely, cerebral vascular accidents that encompass critical areas involved in Broca’s aphasia will sometimes include Broca’s area, thus perpetuating the traditional theory that Broca’s area is the key region in producing Broca’s aphasia.

### Parietal and temporal regions

**A40rv** (overlap 83.3%) is situated in the supramarginal gyrus and is superior to the posterior STG and posterior to the somatosensory representation of tongue and larynx. This region has connectivity with BA44 and the precentral gyrus via the ventral component of the superior longitudinal fasciculus (SLF) III (Hwang et al., 2022; Wang et al., 2016). Notably, this is the only region from the results with connectivity to the right hemisphere per the Brainnetome atlas. **A41/42** (overlap 69.9%) and **TE1.0 and TE2.0** (overlap 80.4%) are components of the human primary auditory cortex, specifically the area near Heschl’s gyrus. This region has massive connectivity with language areas in frontal, temporal, parietal, and insular lobes, as well as interhemispherically. This region also occupies somewhat of a transition cortex between the most inferior portions of the insula as it approaches the superior temporal gyrus and the temporal plane. It is uniquely associated with the cases of chronic Broca’s aphasia. Injury to the temporal lobe is most commonly associated with chronic language disorders (Fridriksson et al., 2015; Dronkers et al., 2004; Binder et al., 1997). Permanent injury to the middle temporal lobe may be responsible for the chronic language deficits observed in Broca’s aphasia.

### White matter pathways

The subtraction map lesion revealed significant disconnection across several major white matter tracts, all of which are critical for language processing and motor functions. The arcuate fasciculus (AF), which is essential for connecting frontal and temporal regions, showed a complete disconnection specifically in participants with Broca’s aphasia (100% index). This disruption indicates a severe impairment in pathways that are crucial for both language comprehension and production, given that the AF links the brain regions responsible for phonological processing and verbal output (Ivanova et al., 2021; Catani et al., 2005). The white matter emanating from the temporal lobe has been reported as injured in cases of post-operative Broca’s aphasia (Andrews et al., 2023). Though the specific tracts in the study by Andrews and colleagues were not reconstructed, the affected regions seem to align with the current findings.

Additionally, the extreme capsule (EC) and the middle longitudinal fasciculus (MdLF) also exhibited complete disconnections (100%), further underscoring the extensive damage to the language processing networks. The EC is a key tract that connects the frontal and temporal lobes and may participate in semantic processing and language comprehension, complementing the dorsal pathway functions typically associated with the AF (Makris and Pandya, 2009). The MdLF, on the other hand, is part of the ventral pathway, connecting temporal regions and playing a significant role in the understanding and production of language (Saur et al., 2008). The complete disconnection of these tracts highlights the profound disruption in the neural networks underlying language, contributing to the significant language deficits observed in Broca’s aphasia. These tracts do not show the same amount of injury in the WNL cohort.

The corticospinal tract (CST), with a near-total disconnection index of 99.96%, may be associated with the significant impairment in motor function often accompanying Broca’s aphasia. This disconnection is not only linked to general motor deficits, like limb paralysis, but also to specific impairments in speech production. The involvement of the CST highlights the overlap between motor and language functions, where damage to this tract can exacerbate the speech production difficulties characteristic of Broca’s aphasia (Duffau, 2015; Duffau et al., 2014).

Similarly, the IFOF which showed a 98.86% disconnection, is involved in integrating visual and language processes. The IFOF, an association tract connecting occipital and frontal regions, plays a role in the semantic and syntactic aspects of language. It passes through the extreme capsule and terminates in the anterior frontal lobe, specifically in the IFG and dorsolateral prefrontal cortex—regions critical for language processing (Catani et al., 2005; Duffau, 2015; Conner et al., 2018). Duffau’s research has consistently emphasized the importance of the IFOF in the language network, demonstrating that its disruption can lead to deficits in language comprehension and production that appear in the complex clinical presentation of Broca’s aphasia (Duffau, 2015; Duffau et al., 2014).

High disconnection indices were also observed in the parietopontine (98.8%) and frontopontine tracts (97.4%), indicating severe disruptions in pathways associated with sensorimotor integration and executive functions. These tracts are essential for coordinating complex behaviors that require the integration of sensory information with motor outputs and higher-order cognitive functions. The extensive disconnection in these pathways may contribute to the broader cognitive and executive function deficits often seen in Broca’s aphasia (Duffau, 2015).

Substantial injury to these tracts was uniquely found in the patients with Broca’s aphasia. In all, these findings underscore the critical role of white matter integrity in supporting language and motor functions. Severe disconnections in these tracts align with previous studies highlighting the importance of both cortical and subcortical pathways in language processing and overall cognitive performance (Ogar et al., 2006; Bates et al., 2003; Ivanova et al., 2021; Griffis et al., 2021; Turken and Dronkers, 2011). Broca’s aphasia is marked by significant disruption in both the ventral and dorsal language pathways, which are crucial for different aspects of language processing. The dorsal pathway, involving the AF, is essential for linking speech sounds to motor actions necessary for speech production, while the ventral pathway, including the extreme capsule, supports semantic processing and language comprehension (Saur et al., 2008; Friederici, 2011). In Broca’s aphasia, disconnections in these pathways—particularly the AF, EC, and MdLF—lead to a widespread breakdown in the neural networks that underlie language (Ivanova et al., 2016; Catani et al., 2005; Dick et al., 2014). This pattern of widespread disconnection across multiple neural pathways underscores the interconnected nature of the brain’s language network and the significant impact that such disruptions can have on both language and cognitive functions.

## Limitations

While our study provides valuable insights, it is important to note that the generalization of findings is limited by the variability in lesion size between the two groups. Additionally, having larger groups and tracking involvement of the outlined areas at different stages of recovery would provide additional insights into their differential role in the symptomatology Broca’s aphasia.

## Conclusion

The current study aimed to investigate the relationship between the neuroanatomical features of specific brain regions and the manifestation of Broca’s aphasia in 39 stroke survivors. Our approach was not limited to Broca’s area, but rather explored broader regions implicated in Broca’s aphasia. Our findings highlight the significant role of the insular regions, particularly the dysgranular portion, in Broca’s aphasia. The unique properties and arrangement of VENs in these regions, suggest they could be involved in efficient speech production. The frontal regions, encompassing the tongue and larynx areas, were significantly injured, underscoring their importance in speech-related motor skills and articulatory control. The temporal lobe injury present uniquely in the cases of Broca’s aphasia reinforces its critical role in language and its association with the chronic symptoms observed in Broca’s aphasia. The use of the Brainnetome atlas (Fan et al., 2016) allowed for a nuanced picture of these regions, integrating cytoarchitectonic details with functional connectivity. Of note, the inferior frontal region traditionally known as Broca’s area was not associated with chronic Broca’s aphasia. Additionally, we report white matter contributions from the left AF, MdLF, CST, and IFOF to cases of Broca’s aphasia. Our study advances the understanding of Broca’s aphasia by demonstrating that the language deficits are not confined to Broca’s area but involve a complex network of brain regions. We hope that understanding the structural features affected by Broca’s aphasia could lead to future therapies that target similar cell populations in perilesional or contralateral regions.

## Supplementary Material

Supplementary Material - Data Sheet

The Supplementary Material for this article can be found online at: https://www.frontiersin.org/articles/10.3389/flang.2025.1496209/full#supplementary-material

## Figures and Tables

**TABLE 1:** Demographic information and mean subtest scores from the Western Aphasia Battery for the cohort with Broca’s aphasia (*N* = 39) and the within normal limits (WNL) cohort (*N* = 41).

Aphasia type	*N*	Age (years)	TPO (months)	Education (years)	Lesion volume (cc)	AQ	Fluency	Aud comp	Repetition	Naming
Broca	39	57.8 ± 11	48 ± 45	14 ± 2	222.76 ± 105.17	40.14 ± 19.6	2.47 ± 1.57	6.94 ± 3.00	2.97 ± 2.4	3.29 ± 2.8
WNL	41	58 ± 12.6	53 ± 65	15.5 ± 2.7	59.82 ± 62.43	97.29 ± 1.8	9.56 ± 0.5	9.91± 0.18	9.76 ± 3.31	9.41 ± 0.34

TPO, time post-onset; AQ, aphasia quotient; Aud Comp, auditory comprehension.

## Data Availability

The datasets presented in this article are not readily available because of privacy limitations for VA patients. Requests to access the datasets should be directed to ivanova@berkeley.edu.

## References

[R1] Allman JM, Tetreault NA, Hakeem AY, Manaye KF, Semendeferi K, Erwin JM, (2011). The von economo neurons in the frontoinsular and anterior cingulate cortex. Ann. N. Y. Acad. Sci 1225, 59–71. doi: 10.1111/j.1749-6632.2011.06011.x 21534993 PMC3140770

[R2] Amunts K, Lepage C, Borgeat L, Mohlberg H, Dickscheid T, Rousseau M-É, (2013). BigBrain: an ultrahigh-resolution 3D human brain model. Science 340, 1472–1475. doi: 10.1126/science.1235381 23788795

[R3] Amunts K, Mohlberg H, Bludau S, and Zilles K (2020). Julich-brain: a 3d probabilistic atlas of the human brain’s cytoarchitecture. Science 369, 988–992. doi: 10.1126/science.abb4588 32732281

[R4] Andrews JP, Cahn N, Speidel BA, Chung JE, Levy DF, Wilson SM, (2023). Dissociation of broca’s area from broca’s aphasia in patients undergoing neurosurgical resections. J. Neurosurg 138, 847–857. doi: 10.3171/2022.6.JNS2297 35932264 PMC9899289

[R5] Baldo JV, Arévalo A, Patterson JP, and Dronkers NF (2013). Grey and white matter correlates of picture naming: evidence from a voxel-based lesion analysis of the Boston Naming Test. Cortex 49, 658–667. 22482693 10.1016/j.cortex.2012.03.001 PMC3613759

[R6] Baldo JV, Wilkins DP, Ogar J, Willock S, and Dronkers NF (2011). Role of the precentral gyrus of the insula in complex articulation. Cortex 47, 800–807. doi: 10.1016/j.cortex.2010.07.001 20691968

[R7] Bates E, Wilson SM, Saygin AP, Dick F, Sereno MI, Knight RT, (2003). Voxel-based lesion-symptom mapping. Nat. Neurosci 6, 448–450. 12704393 10.1038/nn1050

[R8] Binder JR, Frost JA, Hammeke TA, Cox RW, Rao SM, and Prieto T (1997). Human brain language areas identified by functional magnetic resonance imaging. J. Neurosci 17, 353–362. doi: 10.1523/JNEUROSCI.17-01-00353.1997 8987760 PMC6793702

[R9] Brett M, Larson E, Hanke M, Côté M-A, Markiewicz CJ, and Oosterhof NN (2020). Nipy/nibabel: 3.2.0 [Software]. Zenodo. doi: 10.5281/zenodo.4109791

[R10] Broca P-P (1861). Remarques sur le siège de la faculté du langage articulé, suivies d’une observation d’aphémie (perte de la parole). Bulletin et Memoires de la Societe anatomique de Paris 6, 330–357

[R11] Brodmann K (1909). Vergleichende lokalisationslehre der großhirnrinde in ihren prinzipien dargestellt auf grund des zellenbaues. Leipzig: Johann Ambrosius Barth.

[R12] Catani M, Jones DK, and Ffytche DH (2005). Perisylvian language networks of the human brain. Ann. Neurology 57, 8–16. doi: 10.1002/ana.20319 15597383

[R13] Chenausky K, Paquette S, Norton A, and Schlaug G (2020). Apraxia of speech involves lesions of dorsal arcuate fasciculus and insula in patients with aphasia. Neur. Clin. Pract 10, 162–169. doi: 10.1212/CPJ.0000000000000699 PMC7156201 32309035

[R14] Conner AK, Briggs RG, Sali G, Rahimi M, Baker CM, Burks JD, (2018). A connectomic atlas of the human cerebrum—chapter 13: tractographic description of the inferior fronto-occipital fasciculus. Operative Surg 15, S436–S443. doi: 10.1093/ons/opy267 PMC6890527 30260438

[R15] Desikan RS, Ségonne F, Fischl B, Quinn BT, Dickerson BC, Blacker D, (2006). An automated labeling system for subdividing the human cerebral cortex on mri scans into gyral based regions of interest. NeuroImage 31, 968–980. doi: 10.1016/j.neuroimage.2006.01.021 16530430

[R16] Dick AS, Bernal B, and Tremblay P (2014). The language connectome: new pathways, new concepts. Neuroscientist 20, 453–467. doi: 10.1177/1073858413513502 24342910

[R17] Dronkers NF (1996). A new brain region for coordinating speech articulation. Nature 384, 159–161. doi: 10.1038/384159a0 8906789

[R18] Dronkers NF, and Baldo JV (2009). Language: aphasia. Encyclop. Neursci 5, 499–507. doi: 10.1016/B978-008045046-9.01876-3

[R19] Dronkers NF, Ivanova MV, and Baldo JV (2017). What do language disorders reveal about brain–language relationships? From classic models to network approaches. J. Int. Neuropsychol. Soc 23, 741–754. doi: 10.1017/S1355617717001126 29198286 PMC6606454

[R20] Dronkers NF, Wilkins DP, Van Valin RD, Redfern BB, and Jaeger JJ (2004). Lesion analysis of the brain areas involved in language comprehension. Cognition 92, 145–177. doi: 10.1016/j.cognition.2003.11.002 15037129

[R21] Duffau H (2015). Stimulation mapping of white matter tracts to study brain functional connectivity. Nat. Rev. Neurol 11, 255–265. doi: 10.1038/nrneurol.2015.51 25848923

[R22] Duffau H, Moritz-Gasser S, and Mandonnet E (2014). A re-examination of neural basis of language processing: proposal of a dynamic hodotopical model from data provided by brain stimulation mapping during picture naming. Brain Lang 131, 1–10. doi: 10.1016/j.bandl.2013.05.011 23866901

[R23] Fan L, Li H, Zhuo J, Zhang Y, Wang J, Chen L, (2016). The human brainnetome atlas: a new brain atlas based on connectional architecture. Cereb. Cortex 26, 3508–3526. doi: 10.1093/cercor/bhw157 27230218 PMC4961028

[R24] Fridriksson J, Fillmore P, Guo D, and Rorden C (2015). Chronic broca’s aphasia is caused by damage to broca’s and wernicke’s areas. Cereb. Cortex 25, 4689–4696. doi: 10.1093/cercor/bhu152 25016386 PMC4669036

[R25] Friederici AD (2011). The brain basis of language processing: from structure to function. Physiol. Rev 91, 1357–1392. doi: 10.1152/physrev.00006.2011 22013214

[R26] Gajardo-Vidal A, Lorca-Puls DL, Team P, Warner H, Pshdary B, Crinion JT, (2021). Damage to broca’s area does not contribute to long-term speech production outcome after stroke. Brain 144, 817–832. doi: 10.1093/brain/awaa460 33517378 PMC8041045

[R27] Glasser MF, Sotiropoulos SN, Wilson JA, Coalson TS, Fischl B, Andersson JL, (2013). The minimal preprocessing pipelines for the human connectome project. NeuroImage 80, 105–124. doi: 10.1016/j.neuroimage.2013.04.127 23668970 PMC3720813

[R28] Griffis JC, Metcalf NV, Corbetta M, and Shulman GL (2021). Lesion quantification toolkit: a matlab software tool for estimating grey matter damage and white matter disconnections in patients with focal brain lesions. NeuroImage: Clini 30:102639 doi: 10.1016/j.nicl.2021.102639 PMC8053805 33813262

[R29] Grill-Spector K, and Weiner KS (2014). The functional architecture of the ventral temporal cortex and its role in categorization. Nat. Rev. Neurosci 15, 536–548. 24962370 10.1038/nrn3747 PMC4143420

[R30] Hahnloser RHR, Kozhevnikov AA, and Fee MS (2002). An ultra-sparse code underliesthe generation of neural sequences in a songbird. Nature 419, 65–70. doi: 10.1038/nature00974 12214232

[R31] Harris CR, Millman KJ, van der Walt SJ, Gommers R, Virtanen P, Cournapeau D, (2020). Array programming with numpy. Nature 585, 357–362. doi: 10.1038/s41586-020-2649-2 32939066 PMC7759461

[R32] Hubel DH, and Wiesel TN (1997). Ferrier lecture - functional architecture of macaque monkey visual cortex. Proc. Royal Soc. London. Series B. Biol. Sci 198, 1–59. doi: 10.1098/rspb.1977.0085 20635

[R33] Hwang Y-E, Kim Y-B, and Son Y-D (2022). Finding cortical subregions regarding the dorsal language pathway based on the structural connectivity. Front. Hum. Neurosci 16:784340. doi: 10.3389/fnhum.2022.784340 PMC9108242 35585994

[R34] Itabashi R, Nishio Y, Kataoka Y, Yazawa Y, Furui E, Matsuda M, (2016). Damage to the left precentral gyrus is associated with apraxia of speech in acute stroke. Stroke 47, 31–36. doi: 10.1161/STROKEAHA.115.010402 26645260

[R35] Ivanova MV, Isaev DY, Dragoy OV, Akinina YS, Petrushevskiy AG, Fedina ON, (2016). Diffusion-tensor imaging of major white matter tracts and their role in language processing in aphasia. Cortex 85, 165–181. doi: 10.1016/j.cortex.2016.04.019 27289586

[R36] Ivanova MV, Zhong A, Turken A, Baldo JV, and Dronkers NF (2021). Functional contributions of the arcuate fasciculus to language processing. Front. Hum. Neurosci 15:672665 doi: 10.3389/fnhum.2021.672665 PMC8267805 34248526

[R37] Jarvis E (2013). “Evolution of brain pathways for vocal learning in birds and humans,” in Birdsong, Speech, and Language: Exploring the Evolution of Mind and Brain (Boston: MIT Press).

[R38] Jenkinson M, Beckmann CF, Behrens TEJ, Woolrich MW, and Smith SM (2012). FSL. NeuroImage 62, 782–790. doi: 10.1016/j.neuroimage.2011.09.015 21979382

[R39] Kertesz A (2007). Western Aphasia Battery—Revised (WAB-R).

[R40] Kurth F, Zilles K, Fox PT, Laird AR, and Eickhoff SB (2010). A link between the systems: functional differentiation and integration within the human insula revealed by meta-analysis. Brain Struct. Funct 214, 519–534. doi: 10.1007/s00429-010-0255-z 20512376 PMC4801482

[R41] Makris N, and Pandya DN (2009). The extreme capsule in humans and rethinking of the language circuitry. Brain Struct. Funct 213, 343–358. doi: 10.1007/s00429-008-0199-8 19104833 PMC3777634

[R42] McKinney W (2010). “Data structures for statistical computing in python,” in Proceedings of the 9th Python in Science Conference (Austin, TX), 56–61. doi: 10.25080/Majora-92bf1922-00a

[R43] Mohr JP, Pessin MS, Finkelstein S, Funkenstein HH, Duncan GW, and Davis KR (1978). Broca aphasia: pathologic and clinical. Neurology 28, 311–311. doi: 10.1212/WNL.28.4.311 565019

[R44] Morel A, Gallay MN, Baechler A, Wyss M, and Gallay DS (2013). The human insula: architectonic organization and postmortem mri registration. Neuroscience 236, 117–135. doi: 10.1016/j.neuroscience.2012.12.076 23340245

[R45] Mountcastle V (1997). The columnar organization of the neocortex. Brain 120, 701–722. doi: 10.1093/brain/120.4.701 9153131

[R46] Nimchinsky EA, Gilissen E, Allman JM, Perl DP, Erwin JM, and Hof PR (1999). A neuronal morphologic type unique to humans and great apes. Proc. Natl. Acad. Sci. U.S.A 96, 5268–5273. doi: 10.1073/pnas.96.9.5268 10220455 PMC21853

[R47] Ochfeld E, Newhart M, Molitoris J, Leigh R, Cloutman L, Davis C, (2010). Ischemia in broca area is associated with broca aphasia more reliably in acute than in chronic stroke. Stroke 41, 325–330. doi: 10.1161/STROKEAHA.109.570374 20044520 PMC2828050

[R48] Ogar J, Willock S, Baldo J, Wilkins D, Ludy C, and Dronkers N (2006). Clinical and anatomical correlates of apraxia of speech. Brain Lang 97, 343–350. doi: 10.1016/j.bandl.2006.01.008 16516956

[R49] Oishi H, Berezovskii VK, Livingstone MS, Weiner KS, and Arcaro MJ (2024). Inferotemporal face patches are histo-architectonically distinct. Cell Rep 43. 10.1016/j.celrep.2024.114732 PMC12396101 39269905

[R50] Pracar AL, Ivanova MV, Richardson A, and Dronkers NF (2023). A case of pure apraxia of speech after left hemisphere stroke: behavioral findings and neural correlates. Front. Neurol 14:1187399. doi: 10.3389/fneur.2023.1187399 PMC10421996 37576017

[R51] Rorden C (2007). MRIcron

[R52] Saur D, Kreher BW, Schnell S, Kümmerer D, Kellmeyer P, Vry MS, (2008). Ventral and dorsal pathways for language. Proc. Natl. Acad. Sci. U.S.A 105, 18035–18040. doi: 10.1073/pnas.0805234105 19004769 PMC2584675

[R53] Schlaug G, Marchina S, and Wan CY (2011). The use of non-invasive brain stimulation techniques to facilitate recovery from post-stroke aphasia. Neuropsychol Rev 21, 288–301. doi: 10.1007/s11065-011-9181-y 21842404 PMC3176334

[R54] Sperber C, Griffis J, and Kasties V (2022). Indirect structural disconnection-symptom mapping. Brain Struct. Funct 227, 3129–3144. doi: 10.1007/s00429-022-02559-x 36048282

[R55] Turken AU, and Dronkers NF (2011). The neural architecture of the language comprehension network: converging evidence from lesion and connectivity analyses. Front. Syst. Neurosci 5:1. doi: 10.3389/fnsys.2011.00001 21347218 PMC3039157

[R56] Wang X, Pathak S, Stefaneanu L, Yeh FC, Li S, Fernandez-Miranda JC (2016). Subcomponents and connectivity of the superior longitudinal fasciculus in the human brain. Brain Struct. Funct 221, 2075–2092. doi: 10.1007/s00429-015-1028-5 25782434

[R57] Watkins KE, Vargha-Khadem F, Ashburner J, Passingham RE, Connelly A, Friston KJ, (2002). MRI analysis of an inherited speech and language disorder: structural brain abnormalities. Brain 125, 465–478. doi: 10.1093/brain/awf057 11872605

[R58] Watson KK, Jones TK, and Allman JM (2006). Dendritic architecture of the von economo neurons. Neuroscience 141, 1107–1112. doi: 10.1016/j.neuroscience.2006.04.084 16797136

[R59] Weiner KS, and Zilles K (2016). The anatomical and functional specialization of the fusiform gyrus. Neuropsychologia 83, 48–62. 26119921 10.1016/j.neuropsychologia.2015.06.033 PMC4714959

[R60] Wilson SM, Entrup JL, Schneck SM, Onuscheck CF, Levy DF, Rahman M, (2023). Recovery from aphasia in the first year after stroke. Brain 146, 1021–1039. doi: 10.1093/brain/awac129 35388420 PMC10169426

[R61] Yeh F-C, Panesar S, Fernandes D, Meola A, Yoshino M, Fernandez-Miranda JC, (2018). Population-averaged atlas of the macroscale human structural connectome and its network topology. NeuroImage 178, 57–68. doi: 10.1016/j.neuroimage.2018.05.027 29758339 PMC6921501

[R62] Yourganov G, Fridriksson J, Rorden C, Gleichgerrcht E, and Bonilha L (2016). Multivariate connectome-based symptom mapping in post-stroke patients: networks supporting language and speech. J. Neurosci 36, 6668–6679. doi: 10.1523/JNEUROSCI.4396-15.2016 27335399 PMC4916245

[R63] Yushkevich PA, Piven J, Hazlett HC, Smith RG, Ho S, Gee JC, (2006). User-guided 3D active contour segmentation of anatomical structures: Significantly improved efficiency and reliability. NeuroImage 31, 1116–1128. doi: 10.1016/j.neuroimage.2006.01.015 16545965

[R64] Zhong AJ, Baldo JV, Dronkers NF, and Ivanova MV (2022). The unique role of the frontal aslant tract in speech and language processing. NeuroImage: Clinical 34:103020. doi: 10.1016/j.nicl.2022.103020 PMC9095886 35526498

[R65] Zilles K, and Amunts K (2018). Cytoarchitectonic and receptorarchitectonic organization in broca’s region and surrounding cortex. Curr. Opini. Behav. Sci 21, 93–105. doi: 10.1016/j.cobeha.2018.02.011

