# Supplementary material for: The neuroanatomy of Broca’s aphasia
Source: Front Lang Sci. Author manuscript; Available in PMC 2026 Feb 24. (PMC12928379; doi:10.3389/flang.2025.1496209)
Supplement: Supplementary Material - Data Sheet [file NIHMS2148230-supplement-Supplementary_Material_-_Data_Sheet.pdf]

Step-by-Step Explanation of the Subtraction Map (Specific to Broca's Aphasia)

1. **Goal**: Ensure the common lesion areas are not just due to general stroke patterns.

2. **Method**:
   - Compare Broca's aphasia lesions to another group with left-hemisphere strokes but **no aphasia** (called WNL).
   - Create separate lesion overlap maps for the Broca and WNL groups (percentage of patients with lesions in each voxel).
   - Subtract the WNL map from the Broca map.
     - Example: If 70% of Broca patients and 10% of WNL patients had a lesion in the same spot, the subtraction value is **0.6** (70% - 10%).
3. **Thresholding**:
   - To ensure meaningful differences:
     - Find the maximum lesion overlap in Broca's group (**0.97**) and WNL group (**0.42**).
     - Subtract these values to get a threshold of **0.55**.
   - Keep only voxels with a subtraction value of **0.55 or higher**.
   - This isolates areas truly specific to Broca's aphasia, rather than simply those commonly lesioned due to stroke (which would be the case if here we chose a threshold of 0). Note, that if both lesion overlay maps had a similar maximum lesion overlap, then the threshold would have been 0. Thus, thresholding established in this manner allows us to flexibly account for variable lesion size and densities in different cohorts.
4. **Final Steps**:

   - Turn this subtraction map into a binary map (lesion present or not).
   - Use it with the Brainnetome atlas to identify regions uniquely affected in Broca's aphasia.

Summary

- The **common lesion map** shows areas frequently damaged in Broca's aphasia.
- The **subtraction map** isolates areas specific to Broca's aphasia by removing overlap common to all left-hemisphere strokes.
- These maps help pinpoint which brain regions are uniquely involved in Broca's aphasia.
